# Associations among the plasma amino acid profile, obesity, and glucose metabolism in Japanese adults with normal glucose tolerance

**DOI:** 10.1186/s12986-015-0059-5

**Published:** 2016-01-19

**Authors:** Chisa Takashina, Ichizo Tsujino, Taku Watanabe, Shinji Sakaue, Daisuke Ikeda, Asuka Yamada, Takahiro Sato, Hiroshi Ohira, Yoshinori Otsuka, Noriko Oyama-Manabe, Yoichi M. Ito, Masaharu Nishimura

**Affiliations:** First Department of Medicine, Hokkaido University Hospital, North 14, West 5, Kita-ku, Sapporo, 060-8648 Japan; First Department of Medicine, Hokkaido University Graduate School of Medicine, Sapporo, Japan; Department of Human Developmental Science, Faculty of Education, Hokkaido University, Sapporo, Japan; Department of Diagnostic and Interventional Radiology, Hokkaido University Hospital, Sapporo, Japan; Department of Biostatistics, Hokkaido University Graduate School of Medicine, Sapporo, Japan

**Keywords:** Visceral obesity, Oral glucose tolerance test, Insulin secretion, Insulin resistance, Branched-chain amino acid, Glycine, Glutamate, Glutamine

## Abstract

**Background:**

Amino acids (AAs) are emerging as a new class of effective molecules in the etiology of obesity and diabetes mellitus. However, most investigations have focused on subjects with obesity and/or impaired glucose regulation; the possible involvement of AAs in the initial phase of glucose dysregulation remains poorly understood. Furthermore, little attention has been given to possible associations between the pattern/degree of fat deposition and the plasma AA profile. Our objective was therefore to determine the relationships between plasma AA concentrations and the type/degree of obesity and glucose regulation in Japanese adults with normal glucose tolerance.

**Methods:**

Eighty-three subjects with normal glucose tolerance were classified as obese or nonobese and as visceral obesity or nonvisceral obesity. Correlations between the plasma levels of 23 AAs and somatometric measurements, visceral fat area (VFA), subcutaneous fat area (SFA), and 75-g oral glucose tolerance test results were analyzed.

**Results:**

Obesity or visceral obesity was associated with higher levels of branched-chain AAs (isoleucine, leucine, and valine), lysine, tryptophan, cystine, and glutamate but lower levels of asparagine, citrulline, glutamine, glycine, and serine (*p* < 0.04). Age- and gender-adjusted analyses indicated that VFA was positively correlated with tryptophan and glutamate levels, whereas VFA and SFA were negatively correlated with citrulline, glutamine, and glycine levels (*p* < 0.05). The fasting and 2-h plasma glucose levels or the homeostasis model assessment of insulin resistance were positively correlated with valine, glutamate, and tyrosine levels but negatively correlated with citrulline, glutamine, and glycine levels. The homeostasis model assessment for the β-cell function index was positively correlated with leucine, tryptophan, valine, and glutamate levels but negatively correlated with citrulline, glutamine, glycine, and serine levels (*p* < 0.05).

**Conclusions:**

The present study identified specific associations between 10 AAs and the type/degree of obesity, and indices of glucose/insulin regulation, in Japanese adults with preserved glucose metabolism. With the growing concern about the increasing prevalence of obesity and diabetes, the possible roles of these AAs as early markers and/or precursors warrant further investigation.

**Electronic supplementary material:**

The online version of this article (doi:10.1186/s12986-015-0059-5) contains supplementary material, which is available to authorized users.

## Background

Globally, the prevalence of diabetes mellitus (DM), impaired glucose tolerance (IGT), and metabolic syndrome is increasing; these disorders are becoming a major economic and social burden [1]. Insulin resistance plays a central role in the pathogenesis of these disorders, through defects in insulin-mediated glucose uptake and/or glucose release by the liver, muscles, and other peripheral tissues. There is a well-established epidemiological relationship between obesity and insulin resistance. However, this relationship has been investigated less thoroughly in nondiabetic subjects with normal glucose metabolism than in subjects with DM or IGT.

Amino acids (AAs) are emerging as a new class of effective molecules in the etiology of obesity and DM. During a 12-year follow-up study, plasma levels of branched-chain AAs (BCAAs; isoleucine, leucine, and valine), tyrosine, and phenylalanine were reported to predict the development of DM in nondiabetic subjects [2]. Prior clinical studies have also identified significant associations between the plasma levels of specific AAs and body mass index (BMI) [3, 4] or glucose regulation [5]. However, most investigations involved subjects with obesity and/or impaired glucose regulation [6–8]. Thus, the possible involvement of AAs in the initial phase of glucose dysregulation remains poorly understood. Furthermore, little attention has been given to possible associations between the pattern and degree of fat deposition and the plasma AA profile. This is of clinical interest because visceral adipose tissue demonstrates a stronger association with metabolic disturbances and cardiovascular risks than subcutaneous adipose tissue [9, 10]. Finally, most related studies involved mainly non-Asian subjects [4, 5, 11], who differ physically and metabolically from the Asian population [12, 13]. Indeed, a community-based observational study documented that Asians have a lower BMI but a higher percentage of body fat than Caucasians [14]. Based on these and other publications from around the world [15, 16], a definition for obesity specific to the Japanese population was proposed as BMI ≥ 25 kg/m^2^ [17], which differed from that for Europeans (BMI ≥ 30 kg/m^2^).

The aim of the present study was to investigate the relationships between the plasma AA profile and the type/degree of obesity as well as glucose and insulin regulation, in normoglycemic Japanese adults. Using a detailed analysis of the indices of insulin resistance, sensitivity, and secretion derived from the 75-g oral glucose tolerance test (75-g OGTT), this study identified significant associations between AAs and the development of aberrant glucose regulation in nondiabetic Japanese adults.

## Methods

### Subjects

Ninety-four healthy Japanese volunteers aged 20–60 years were recruited between March 2009 and March 2010 from the medical students, interns, employees, and paramedical staff of Hokkaido University Hospital. We excluded subjects with a history of DM, chronic kidney disease, or liver disease, and those taking medications that could affect glucose metabolism, such as angiotensin-converting enzyme inhibitors, angiotensin II receptor inhibitors, statins, quinolones, antimicrobial agents, and any drug that presents hormonal effects. Finally, those unable to undergo magnetic resonance imaging (MRI) for any reason were excluded. This study was approved by the Ethical Committee on Human Experiments of Hokkaido University Graduate School of Medicine (MED08-003), and written informed consent was obtained from all subjects.

All subjects underwent somatometry, blood sampling, 75-g OGTT, and MRI within a week of their enrollment, during which there was no significant change in their lifestyle, including their dietary intake, or their physical condition.

### Somatometry

Height and body weight (BW) were measured using standard methods on the same day as the blood sampling for plasma AA measurements and the 75-g OGTT. Waist circumference (WC) was measured in the standing position, at the umbilical level, after expiration during natural breathing. BMI was calculated using the following formula: BW (kg)/(height (m))^2^. The subjects were divided into two groups based on the BMI cutoff value for obesity determined for the Japanese population (BMI ≥ 25 kg/m^2^) [17].

### The 75-g OGTT

The subjects were tested using the standard 75-g OGTT procedure [18]. Briefly, the subjects were fasted from 11 pm on the previous day until 9 am, when they were given 300 ml of aqueous solution containing 75 g of glucose to drink within 5 min. Blood samples were collected from an antecubital vein immediately before ingestion of the solution (fasting plasma glucose [FPG]) and 30, 60, 90, and 120 min after ingestion. The samples were collected in tubes containing fluoride and maintained at 4 °C until they were centrifuged, within 2 h, to measure the plasma glucose (PG) and plasma insulin levels. The results were interpreted according to the World Health Organization definitions: normal glucose tolerance (NGT; FPG < 110 mg/dl and 2-h PG < 140 mg/dl), impaired fasting glucose (FPG 110–125 mg/dl and 2-h PG < 140 mg/dl), IGT (FPG < 126 mg/dl and 2-h PG 140–199 mg/dl), and DM (FPG ≥ 126 mg/dl or 2-h PG ≥ 200 mg/dl) [19]. Insulin resistance was determined using the homeostasis model assessment of insulin resistance (HOMA-IR) index, calculated as follows: FPG (mg/dl) × fasting plasma insulin (μU/ml) / 405 [20]. Insulin sensitivity was assessed by calculating the oral glucose insulin sensitivity (OGIS) index using a spreadsheet downloaded from the OGIS website [21, 22]. Insulin secretion was measured using the homeostasis model assessment of β-cell function index (HOMA-β), calculated as follows: (fasting plasma insulin (μU/ml) × 360)/(FPG (mg/dl) − 63) [20]. Insulin secretion was also expressed in terms of the insulinogenic index (II): 0–30 min insulin increment (μU/ml)/0–30 min PG increment (mg/dl) [23].

### Measurement of plasma AA concentrations

Separate blood specimens were collected from the antecubital vein into heparinized tubes at the time the fasting blood specimens were obtained for the 75-g OGTT. The plasma was separated at 4 °C and stored at −80 °C until the measurements were made. The plasma was deproteinized in a final concentration of 80 % acetonitrile, and the plasma AAs were identified and quantified using high-performance liquid chromatography (L-8500; Hitachi, Ltd., Tokyo) after derivatization (SRL, Inc., Tokyo) [24, 25].

Twenty-three compounds were analyzed in each plasma sample to cover the main essential AAs acquired from the diet (histidine, isoleucine, leucine, lysine, methionine, phenylalanine, threonine, tryptophan, and valine) as well as the nonessential AAs (alanine, arginine, asparagine, α-aminobutyric acid, citrulline, cystine, glutamate, glutamine, glycine, ornithine, proline, serine, taurine, and tyrosine). Of these, the BCAAs are isoleucine, leucine, and valine.

### Evaluation of visceral and subcutaneous fat by MRI

The MRIs were performed with an Achieva 1.5 T (Philips Medical System, Best, The Netherlands) equipped with a gantry coil. Coronal, sagittal, and long-axis scout images were obtained, covering the whole body from the cervix to the pelvis. Transverse axial fat imaging was then performed during repeated breath-holds, using a T1-turbo field echo (TFE) sequence with spectral attenuated inversion recovery (SPAIR) for water suppression. The acquisition parameters were as follows: field of view, 450 × 310 mm; (repetition time)/(echo time), 3.6/1.7 ms; acquisition matrix, 128 × 100; slice thickness, 6 mm; flip angle, 10°; and TFE factor, 30. For SPAIR, the repetition time was 206 ms, and the inversion time was 80 ms.

The areas of visceral and subcutaneous fat were measured using commercially available software (FatChecker, VOX-BASE; J-MAC system, Sapporo, Japan). In brief, a transaxial image at the level of the umbilicus was selected from the stack of abdominal short-axis images. In this image, fat tissue was distinguished from the other tissues (muscles and bones) based on the minimum and maximum signal intensities of each tissue, calculated using an approximation of the Gaussian distribution of the overall histogram. The boundaries between the subcutaneous fat and visceral fat were automatically traced using the software’s tracking operation. The subcutaneous fat and visceral fat were identified as the regions outside and inside of these boundaries, respectively. Each fat region identified with this method was visually confirmed, and the border manually corrected as needed. The subcutaneous fat area (SFA) and visceral fat area (VFA) were calculated from the number of pixels in the histograms and the pixel size. The subjects were divided into two groups based on the widely used VFA cutoff value of 100 cm^2^: the visceral obesity group (VFA ≥ 100 cm^2^) and the nonvisceral obesity group (VFA < 100 cm^2^) [17].

### Data analysis

All values are presented as mean ± SD. The obese and nonobese groups, and the visceral obesity group and nonvisceral obesity groups, were compared in terms of demographic parameters, somatometric indices, 75-g OGTT results, SFA, VFA, and plasma AA concentrations using chi-square tests or unpaired *t*-tests, as appropriate.

Plasma AA concentrations were also compared between men and women using an unpaired *t*-test.

Associations between plasma AA concentrations and other variables (age, BW, BMI, WC, VFA, SFA, FPG, 2-h PG, HOMA-IR, OGIS, HOMA-β, and II) were assessed by Pearson’s correlation analysis and also by multivariate logistic regression analysis adjusted for age and gender.

The reproducibility of the SFA and VFA measurements was determined using randomly selected MRI images from 10 subjects, measured by two investigators (AY and CT) separated by an interval of at least 2 weeks. The reproducibility analysis was conducted using Bland–Altman plots and intraclass correlation coefficients. The investigators were unaware of the somatometric data or the blood test results.

All statistical analyses were conducted using JMP software version 10, and *p* values <0.05 were considered statistically significant.

## Results

Among the 94 adult volunteers, 11 subjects were excluded from the analysis because the 75-g OGTT test indicated that they had DM (*n* = 3) or IGT (*n* = 8). The remaining 83 subjects, 66 men and 17 women all with NGT, were divided into two groups based on their BMI: obese (*n* = 10) or nonobese (*n* = 73). These subjects were also divided into two groups based on their VFA: visceral obesity (*n* = 18) or nonvisceral obesity (*n* = 65). The two pairs of groups were compared in terms of demographic, somatometric, 75-g OGTT, and MRI data (Table 1). The obese group presented significantly higher somatometric parameters (BW, BMI, and WC), FPG, insulin resistance (HOMA-IR), insulin secretion (II), and SFA, but a lower insulin sensitivity (OGIS) than the nonobese group (all *p* < 0.001). The visceral obesity group included significantly more men than the nonvisceral obesity group, as well as significantly higher age, somatometric parameters (BW, BMI, and WC), and II (all *p* ≤ 0.017).

**Table 1:** Somatometric measurements and the results of 75-g oral glucose tolerance test and magnetic resonance imaging

	Overall (*n* = 83)	Obese (*n* = 10)	Nonobese (*n* = 73)	*p* values ^a^	Visceral obesity (*n* = 18)	Nonvisceral obesity (*n* = 65)	*p* values^b^
Gender (male/female)	66/17	10/0	56/17	0.114	18/0	48/17	**0.017**
Age (years)	34.0 ± 5.7	35.2 ± 3.8	33.8 ± 5.9	0.487	37.1 ± 7.0	33.2 ± 5.0	**0.009**
Somatometric measurements							
Body weight (kg)	65.5 ± 9.9	79.5 ± 8.5	63.5 ± 8.5	**<0.001**	72.0 ± 8.2	63.7 ± 9.6	**0.001**
Body mass index (kg/m^2^)	22.5 ± 2.4	26.7 ± 2.3	21.9 ± 1.8	**<0.001**	24.1 ± 2.6	22.0 ± 2.2	**0.001**
Waist circumference (cm)	78.8 ± 7.2	88.9 ± 5.8	77.4 ± 6.2	**<0.001**	84.8 ± 6.7	77.2 ± 6.4	**<0.001**
75 g-oral glucose tolerance test							
Fasting plasma glucose (mg/dl)	91.7 ± 8.1	100.7 ± 5.2	90.4 ± 7.6	**<0.001**	91.9 ± 9.4	91.6 ± 7.7	0.868
2-h plasma glucose (mg/dl)	104.3 ± 16.1	110.3 ± 17.2	103.4 ± 15.9	0.209	108.1 ± 19.2	103.2 ± 15.1	0.255
Insulin resistance (HOMA-IR)	1.2 ± 0.8	2.1 ± 1.6	1.1 ± 0.5	**<0.001**	1.5 ± 1.3	1.2 ± 0.6	0.067
Insulin sensitivity (OGIS)	446.6 ± 47.1	394.0 ± 52.0	453.8 ± 41.8	**<0.001**	436.6 ± 65.4	449.4 ± 40.9	0.312
Insulin secretion (HOMA-β)	71.3 ± 37.8	78.8 ± 53.6	70.3 ± 35.4	0.507	82.4 ± 45.7	68.2 ± 35.0	0.159
Insulin secretion (II)^c^	0.3 ± 0.2	0.5 ± 0.4	0.3 ± 0.1	**<0.001**	0.4 ± 0.4	0.3 ± 0.1	**0.004**
Magnetic resonance imaging-derived fat area							
Visceral fat area (cm^2^)	77.1 ± 34.3	88.0 ± 39.2	75.6 ± 33.6	0.285	127.6 ± 27.1	63.1 ± 19.8	**<0.001**
Subcutaneous fat area (cm^2^)	122.9 ± 42.5	176.8 ± 51.2	115.5 ± 35.6	**<0.001**	136.4 ± 43.5	119.2 ± 41.8	0.129

Data are mean ± SD. obese: BMI ≥ 25, nonobese: BMI < 25; visceral obesity: visceral fat area ≥ 100 cm^2^, nonvisceral obesity: visceral fat area < 100 cm^2^

HOMA-β, homeostasis model assessment for β-cell function index; HOMA-IR, homeostasis model assessment for insulin resistance; II, insulinogenic index; OGIS, oral glucose insulin sensitivity. ^a^obese vs nonobese, ^b^Visceral obesity vs Nonvisceral obesity, ^c^ *n* = 81 (Data set from 2 subjects were excluded due to unphysiological glucose/insulin levels. Bold numbers indicate significant differences between the two groups with p <0.05

Table 2 shows that, for the essential AAs, obesity and visceral obesity were both associated with higher plasma concentrations of the three BCAAs (isoleucine, leucine, and valine; all *p* ≤ 0.033). Lysine levels were higher in the obese group (*p* = 0.006), and tryptophan levels were higher in the visceral obesity group (*p* = 0.004). In contrast, the analysis revealed remarkably different impacts of obesity and visceral obesity on the nonessential AA profile. Obesity was associated with higher levels of cystine and glutamate but lower levels of glycine. Visceral obesity was associated with higher levels of glutamate but lower levels of asparagine, citrulline, glutamine, glycine, and serine.

**Table 2:** Plasma amino acid concentrations in the obese, nonobese, visceral obese, and nonvisceral obese groups

	Overall	Obese	Nonobese	*p* values^a^	Visceral obesity	Nonvisceral obesity	*p* ^b^
(nmol/ml)	(*n* = 83)	(*n* = 10)	(*n* = 73)		(*n* = 18)	(*n* = 75)	values^a^
Essential AAs							
Histidine	85.42 ± 9.71	88.02 ± 9.95	85.06 ± 9.69	0.370	84.83 ± 7.94	85.58 ± 10.19	0.774
Isoleucine	64.07 ± 12.09	72.94 ± 12.74	62.86 ± 11.57	**0.013**	70.99 ± 10.59	62.16 ± 11.85	**0.005**
Leucine	125.07 ± 20.71	139.34 ± 15.73	123.12 ± 20.62	**0.019**	136.51 ± 15.93	121.90 ± 20.85	**0.007**
Lysine	187.46 ± 26.41	208.75 ± 23.59	184.55 ± 25.56	**0.006**	186.83 ± 21.20	187.64 ± 27.82	0.909
Methionine	26.51 ± 3.71	26.69 ± 3.89	26.48 ± 3.71	0.871	25.81 ± 3.97	26.70 ± 3.65	0.370
Phenylalanine	57.67 ± 6.79	59.54 ± 4.86	57.41 ± 7.00	0.356	59.13 ± 8.34	57.26 ± 6.31	0.305
Threonine	129.23 ± 26.66	126.43 ± 19.06	129.61 ± 27.62	0.726	122.77 ± 23.68	131.02 ± 27.33	0.248
Tryptophan	56.04 ± 10.08	53.93 ± 6.27	56.33 ± 10.49	0.484	61.94 ± 15.43	54.40 ± 7.38	**0.004**
Valine	231.17 ± 30.90	261.89 ± 27.62	226.96 ± 29.03	**<0.001**	244.87 ± 25.78	227.38 ± 31.30	**0.033**
Nonessential AAs							
Alanine	342.61 ± 79.73	359.03 ± 99.02	340.36 ± 77.28	0.491	349.52 ± 83.12	340.70 ± 79.33	0.681
Arginine	66.10 ± 16.33	70.04 ± 17.12	65.56 ± 16.27	0.420	64.26 ± 10.86	66.62 ± 17.58	0.591
Asparagine	46.49 ± 6.19	46.17 ± 7.03	46.53 ± 6.12	0.865	42.73 ± 5.92	47.53 ± 5.89	**0.003**
α-ABA	20.11 ± 5.50	22.01 ± 5.98	19.85 ± 5.42	0.247	20.17 ± 6.39	20.10 ± 5.28	0.963
Citrulline	28.34 ± 6.20	26.23 ± 6.23	28.63 ± 6.19	0.254	25.57 ± 4.99	29.11 ± 6.32	**0.031**
Cystine	36.67 ± 6.41	41.47 ± 7.06	36.02 ± 6.08	**0.011**	37.59 ± 7.53	36.42 ± 6.11	0.497
Glutamate	57.93 ± 19.19	70.06 ± 17.04	56.27 ± 18.97	**0.032**	71.88 ± 24.25	54.07 ± 15.68	**<0.001**
Glutamine	524.27 ± 55.46	514.17 ± 80.48	525.66 ± 51.75	0.542	495.84 ± 60.17	532.15 ± 51.86	**0.013**
Glycine	220.56 ± 34.87	197.88 ± 41.43	223.67 ± 33.00	**0.027**	193.69 ± 30.16	228.00 ± 32.51	**<0.001**
Ornithine	80.34 ± 16.80	86.87 ± 14.91	79.44 ± 16.94	0.192	81.85 ± 15.20	79.92 ± 17.30	0.669
Proline	147.67 ± 39.39	141.67 ± 32.09	148.50 ± 40.40	0.610	151.29 ± 33.02	146.67 ± 41.15	0.662
Serine	116.31 ± 19.30	112.34 ± 15.82	116.86 ± 19.76	0.491	104.54 ± 16.36	119.57 ± 18.87	**0.003**
Taurine	74.99 ± 18.72	69.79 ± 15.03	75.70 ± 19.15	0.352	71.52 ± 15.75	75.95 ± 19.47	0.377
Tyrosine	61.26 ± 9.71	66.44 ± 13.47	60.55 ± 8.97	0.072	62.76 ± 9.47	60.84 ± 9.80	0.461

Data are mean ± SD. obese: BMI ≥ 25, nonobese: BMI < 25; visceral obesity: visceral fat area ≥ 100 cm^2^, nonvisceral obesity: visceral fat area < 100 cm^2^

*AA* amino acid, *α-ABA* α-aminobutyric acid, *BMI* body mass index

^a^Obese vs Nonobese, ^b^Visceral obesity vs Nonvisceral obesity. Bold numbers indicate significant differences between the two groups with p <0.05

Comparison of the AA concentrations between men and women showed that those of 11 AAs were higher in men than in women, but serine levels were lower in men than in women (Additional file 1: Table S1). There were significant correlations between age and the plasma concentrations of four AAs (Additional file 2: Table S2). In a subgroup analysis involving only the 66 men, 7 of the 10 AAs that exhibited significantly different concentrations in the overall analysis on 83 subjects still showed different plasma concentrations between visceral and nonvisceral obesity groups (Additional file 3: Table S3). The men were further divided into two subsets (each *n* = 33) according to whether they were aged above or below the median age of 35. A comparison of AA concentrations showed that significant differences existed for two AAs (glutamate and glutamine) in the older subset (>35 years) and for eight AAs in the younger subset (Additional file 4: Table S4). Overall, these results suggested significant impacts of gender and age on the AA concentrations. At the same time, they also indicated the associations between AA concentrations and visceral obesity because significant differences of the concentrations of some AAs remained between the subjects with or without visceral obesity in the age- and gender-adjusted analyses.

The associations between AAs and the glucose regulation of the NGT subjects was investigated by Pearson’s correlation analysis (Table 3, Additional file 5: Table S5) and by age- and gender-adjusted logistic regression analysis (Table 4, Additional file 6: Table S6) of the plasma AA concentrations of all 83 subjects and the somatometric parameters (BW, BMI, and WC), body fat areas (VFA and SFA), and the indices derived from the 75-g OGTT (HOMA-IR, OGIS, HOMA-β, and II).

**Table 3:** Correlations of plasma amino acid concentrations with indices of obesity and glucose metabolism

	Body weight	BMI	WC	VFA	SFA	FPG	2-h PG	HOMA-IR	OGIS	HOMA-β	II
Essential AAs											
Histidine	—	—	—	—	—	—	—	—	—	—	—
Isoleucine	0.413	0.305	0.345	0.332	—	—	—	—	—	—	—
Leucine	0.488	0.300	0.361	0.413	—	—	—	—	—	—	—
Lysine	0.307	—	0.290	—	—	0.236	—	—	−0.257	—	—
Methionine	—	—	—	—	—	—	—	—	—	—	—
Phenylalanine	0.276	—	—	—	—	—	—	—	—	—	—
Threonine	—	—	—	—	—	—	—	—	—	—	—
Tryptophan	—	—	—	0.289	—	—	—	—	—	—	—
Valine	0.539	0.387	0.482	0.397	—	0.254	—	0.219	−0.297	—	—
Nonessential AAs											—
Alanine	—	—	—	—	—	—	—	—	—	—	—
Arginine	—	—	—	—	—	—	—	—	—	—	—
Asparagine	—	—	—	—	—	—	—	—	—	—	—
α-ABA	—	—	—	—	—	—	—	—	—	—	—
Citrulline	—	—	−0.242	—	−0.395	—	—	−0.291	—	−0.375	—
Cystine	0.276	—	0.285	—	—	0.236	—	—	—	—	—
Glutamate	0.548	0.443	0.528	0.568	—	0.439	0.302	0.292	−0.458	—	—
Glutamine	−0.241	−0.289	−0.326	−0.272	−0.396	—	−0.220	−0.398	—	−0.311	—
Glycine	−0.333	−0.418	−0.426	−0.426	−0.294	—	−0.247	−0.317	0.303	−0.231	—
Ornithine	—	—	—	—	—	—	—	—	—	—	—
Proline	—	—	—	—	—	—	—	—	—	—	—
Serine	−0.368	−0.273	−0.351	−0.379	—	—	—	—	0.258	—	—
Taurine	—	—	—	—	—	−0.240	—	—	—	—	—
Tyrosine	0.335	—	0.314	0.252	—	0.269	—	—	−0.327	—	—

Each number indicates a correlation coefficient for two variables that showed statistically significant correlation by Pearson’s analysis. *AA* amino acid, *α-ABA* α-aminobutyric acid, *BMI* body mass index, *FPG* fasting plasma glucose, *HOMA-β* homeostasis model assessment for β-cell function index, *HOMA-IR* homeostasis model assessment of insulin resistance, *II* insulinogenic index, *OGIS* oral glucose insulin sensitivity, *SFA* subcutaneous fat area, *VFA* visceral fat area, *WC* waist circumference, *2-h PG* 2-h plasma glucose

**Table 4:** Age- and gender-adjusted correlations of plasma amino acid concentrations with obesity and glucose metabolism indices

	Body weight	BMI	WC	VFA	SFA	FPG	2-h PG	HOMA-IR	OGIS	HOMA-β	II
Essential AAs											
Histidine	—	—	—	—	—	—	—	—	—	—	—
Isoleucine	—	—	—	—	—	—	—	—	—	—	—
Leucine	—	—	—	—	—	—	—	—	—	0.288	—
Lysine	—	—	—	—	—	—	—	—	—	—	—
Methionine	—	—	—	—	—	—	—	—	—	—	—
Phenylalanine	—	—	—	—	—	—	—	—	—	—	—
Threonine	—	—	—	—	—	—	—	—	—	—	—
Tryptophan	—	—	—	0.206	—	—	—	—	—	0.259	—
Valine	0.202	0.247	0.296	—	0.348	—	—	0.380	-	0.272	0.419
Nonessential AAs											
Alanine	—	—	0.184	—	—	—	—	—	—	—	—
Arginine	—	—	—	—	—	—	—	—	—	—	—
Asparagine	—	—	—	—	—	—	—	—	—	—	—
α-ABA	—	—	—	—	—	—	—	—	—	—	—
Citrulline	−0.183	−0.286	−0.358	−0.285	−0.382	—	—	−0.296	-	−0.323	−0.307
Cystine	—	—	—	—	—	—	—	—	—	—	—
Glutamate	0.247	0.327	0.352	0.362	0.401	0.395	0.310	0.436	−0.377	0.243	0.403
Glutamine	−0.265	−0.308	−0.282	−0.233	−0362	—	—	−0395	—	−0.341	−0.434
Glycine	−0.221	−0.364	−0.316	−0.311	−0.311	—	—	−0.325	0.226	−0.309	−0.380
Ornithine	—	—	—	—	—	—	—	—	—	—	—
Proline	—	—	—	—	—	—	—	—	—	—	—
Serine	—	—	—	—	—	—	—	—	—	−0.280	—
Taurine	—	—	—	—	—	—	—	—	—	—	—
Tyrosine	0.164	—	—	—	—	—	—	0.226	−0.238	—	—

Each number indicates a standardized coefficient for two variables that showed significant association in the age- and gender-adjusted multiple logistic regression analysis. *AA* amino acid, *α-ABA* α-aminobutyric acid, *BMI* body mass index, *FPG* fasting plasma glucose, *HOMA-β* homeostasis model assessment for β-cell function index, *HOMA-IR* homeostasis model assessment of insulin resistance, *II* insulinogenic index, *OGIS* oral glucose insulin sensitivity, *SFA* subcutaneous fat area, *VFA* visceral fat area, *WC* waist circumference, *2-h PG* 2-h plasma glucose

Regarding the essential AAs, the age- and gender-adjusted analysis showed that the levels of tryptophan and valine correlated positively with the somatometric parameters (BW, BMI, and WC), VFA, and/or SFA. Leucine, tryptophan, and valine levels exhibited positive correlations with insulin resistance (HOMA-IR) or insulin secretion (HOMA-β and II). Notably, none of the 75 g-OGTT parameters were associated with the concentrations of the remaining six essential AAs.

The plasma concentrations of nonessential AAs exhibited positive or negative correlations with the other parameters. The most widespread associations were identified for glutamate and glutamine. Plasma glutamate levels positively correlated with all the somatometric parameters, BW, BMI, WC, VFA and SFA, glucose levels (FPG and 2 h-PG), insulin resistance (HOMA-IR), and insulin secretion (HOMA-β and II) but negatively correlated with insulin sensitivity (OGIS). Interestingly, plasma glutamine levels negatively correlated with all the somatometric parameters and the 75 g-OGTT parameters (except FPG, 2 h-PG, and OGIS). Plasma glycine levels negatively correlated with the somatometric parameters (BW, BMI, and WC), VFA and SFA but correlated positively with insulin sensitivity (OGIS) and negatively with insulin resistance (HOMA-IR) and insulin secretion (HOMA-β and II). Citrulline levels negatively correlated with BW, BMI, WC, VFA and SFA, insulin resistance (HOMA-IR), and insulin secretion (HOMA-β and II). Conversely, tyrosine levels positively correlated with BW and HOMA-IR but negatively correlated with insulin sensitivity (OGIS). Finally, alanine levels correlated positively with WC and serine levels correlated negatively with HOMA-β.

The reproducibility of the fat area measurements performed by two independent observers was validated by using Bland–Altman plots. Only small intraobserver (VFA: −0.4 ± 5.9 (2SD), SFA: −0.1 ± 1.9) and interobserver (VFA: −1.5 ± 8.4, SFA: 0.0 ± 2.0) differences were detected. The intraclass correlation coefficients for intraobserver reproducibility were 1.000 for VFA and SFA, and for interobserver reproducibility, these were 0.9995 for VFA and 1.000 for SFA.

## Discussion

The present study, exclusively involving Japanese adults with NGT, demonstrated that the plasma levels of specific AAs were associated with parameters of obesity, and/or indices of insulin resistance, sensitivity, and secretion derived from the 75-g OGTT. These findings support possible associations of AAs with the type and degree of obesity and glucose regulation in the Asian population with preserved glucose metabolism.

An important result of the present study was the establishment of a link between plasma AA levels and type of obesity: visceral or subcutaneous. We demonstrated that tryptophan is positively associated only with VFA, whereas valine, one of the three BCAAs, is positively associated only with SFA. These data suggest that the known associations between obesity and certain AAs may be derived from a distinct link with visceral fat and/or subcutaneous fat. A previous study on Japanese adults reported similar results; however, the participants did not undergo 75-g OGTT, and those with DM or IGT were not excluded [26]. Conversely, citrulline, glutamine, and glycine negatively correlated with both VFA and SFA, and glutamate positively correlated with both VFA and SFA. These AAs may have broad associations with the overall deposition of fat, rather than with the pattern of fat deposition. Importantly, 88 % of the subjects were considered nonobese (BMI < 25 kg/m^2^), and 78 % of them were considered not to have significant visceral obesity (VFA < 100 cm^2^). Thus, this study demonstrated a significant link between AAs in the blood and body fat in nonobese and nonviscerally obese adult Asians.

Three possibilities could contribute to an explanation of the association between plasma AA levels and the type/degree of obesity identified in the present study: 1. plasma AA levels affect the type/degree of obesity; 2. different obesity types affect plasma AA levels; and 3. there are confounding factors that affect both plasma AA levels and obesity.

The first possibility relates to the appetite-regulating effects of certain AAs. Glutamate is a substrate in the formation of gamma aminobutyric acid, a known appetite enhancer [27]. In contrast, glutamine increases the secretion of glucagon-like peptide (GLP)-1, which is an appetite suppressor [28]. Therefore, the positive correlation of glutamate and the negative associations of glutamine with the obesity-related indices may be related to the regulation of food intake. In addition, an animal study showed that glycine reduces visceral fat through the oxidization of free fatty acids in adipose tissue [29]. This function is consistent with the negative correlation between high plasma glycine levels and BMI, VFA, and SFA.

The second possibility is that the two types of obesity could affect the AA levels. For instance, a study on rats and human specimens revealed that BCAAs including valine are catabolized in visceral and subcutaneous adipose tissue [30]. Also, a rat model of obesity exhibited enhanced protein degradation and reduced catabolic activities toward BCAAs, which could increase their plasma levels [31]. However, a different catabolism of AAs in visceral and subcutaneous fat tissue has not been clearly demonstrated, indicating the need for further research to support this speculation.

The third possibility is that confounding factors, such as dietary intake or exercise, may affect AA levels and obesity. For example, glutamate is abundant in wheat protein, which is the second highest dietary carbohydrate in Japan. In some subjects, excess wheat intake may have caused both an increase in the levels of glutamate and an increase in BMI, VFA, and SFA. Conversely, prolonged submaximal exercise has been reported to reduce plasma leucine concentration and BMI [32]. This finding may be consistent with the significantly lower concentration of leucine in the nonobese group compared with the obese group.

With regard to the link between AAs and the indices of glucose regulation, the present study identified positive correlations of glutamate concentration with FPG and 2-h PG in the Japanese NGT subjects. This finding is partially supported by prior studies in which plasma glutamate, valine, and tyrosine levels were elevated in patients with metabolic syndrome [6] or type 2 DM [33, 34]. These data support the presence of a mechanistic link between an altered plasma AA profile and aberrant glucose regulation, even in subjects with a preserved glucose metabolism. A recent prospective study on 2,422 normoglycemic individuals demonstrated that the plasma levels of five AAs (isoleucine, leucine, valine, tyrosine, and phenylalanine) predicted the development of diabetes during a 12-year follow-up [2]. These results and ours support a possible role for AA metabolism early in the development of DM.

In the present study, the plasma levels of six AAs exhibited relationships with the index of insulin resistance (HOMA-IR) and/or the index of insulin sensitivity (OGIS). Glutamate and tyrosine levels were positively correlated with HOMA-IR and negatively correlated with OGIS, whereas the opposite was the case for glycine. Single associations were also identified for valine, citrulline, and glutamine (with HOMA-IR). This complex network of interactions between AAs and insulin regulation is partially supported by a recent study conducted on Japanese type 2 DM patients, in which the levels of the BCAAs (isoleucine, leucine, and valine), alanine, glutamate, proline, and tyrosine positively correlated with HOMA-IR [8]. The fact that a positive correlation of valine, glutamate, and tyrosine with HOMA-IR was already present in our NGT subjects allows a speculation that these AAs may be associated with the initial phase of the development of glucose dysregulation. Oral glutamate supplements have been reported to impair insulin sensitivity and to induce the development of diabetes in animal studies [35]. In addition, feeding animals with BCAAs induced insulin resistance, along with the chronic phosphorylation of mTOR, JNK, and IRS1_Ser307_ [3]. Also, valine is a glycogenic AA and, in a rat study, its oral administration increased the PG level [36]. These data support an incremental effect of glutamate and valine on blood glucose levels. Finally, as tyrosine is a precursor of catecholamines, the accumulation of this AA could promote an increase in blood glucose level through the anti-insulin action of catecholamines. Collectively, prior studies and ours suggest that specific AAs may indirectly induce an increase in blood glucose level through the stimulation of insulin resistance.

Glutamine has been reported to induce the secretion of GLP-1 in healthy and diabetic subjects [28], suggesting that this AA reduces PG levels through the stimulatory effect of GLP-1 on insulin secretion. Also, leucine synergistically stimulates insulin secretion and lowers blood glucose when ingested with glucose in healthy humans [37]. In addition, in healthy first-degree relatives of type 2 DM patients, oral glycine has been reported to increase insulin secretion without affecting insulin sensitivity [11]. Also, in another human study, single oral administration of glycine significantly stimulated insulin secretion whereas, when given together with glucose, it augmented insulin action and attenuated the increase of blood glucose levels [38]. Thus, further studies are required to clarify the mechanisms by which AAs affect glucose/insulin regulation.

WC is a basic and widely used somatometric index of visceral fat adiposity. In Japan, the cutoff levels for visceral fat obesity are 85 cm for men and 90 cm for women, because these cutoff levels correlate well with a VFA of 100 cm^2^ [12]. Indeed, in our NGT subjects, WC was greater in the visceral obesity group than in the nonvisceral obesity group. The correlation analysis indicated that WC was correlated with the plasma levels of six AAs, which is a greater number than for BMI (five AAs) and VFA (five AAs). These suggest that WC is not only a marker of visceral obesity but also a noninvasive parameter that could be used in future studies on possible associations between AAs and obesity and/or glucose regulation.

Our study had some limitations. First, we did not collect lifestyle data for subjects; thus, we could not exclude the possibility that factors related to lifestyle, such as dietary intake and/or physical activity, affected the subjects’ plasma AA profiles, obesity, and glucose metabolism [39]. Second, only one participant had a BMI ≥ 30 kg/m^2^, and so the findings cannot be extrapolated to a population with higher BMI. Similarly, due to the racial variation in the relation between AAs and insulin resistance/sensitivity, the results need to be interpreted cautiously when extrapolated to other races [40]. Finally, the observational nature of the present study did not allow a direct investigation of the causal relationships between the parameters. However, only Japanese adults with preserved glucose metabolism were included in the study following careful selection using the 75-g OGTT. Thus, the results obtained are meaningful for future basic and clinical research on the potential role of AAs in the pathogenesis and/or prevention of obesity and DM.

## Conclusions

The present study identified specific associations between 10 AAs and the type and degree of obesity, or indices of glucose/insulin regulation, in Japanese adults with preserved glucose metabolism. With the growing concern about the increasing prevalence of obesity and DM, the possible roles of these AAs as early markers and/or precursors of these conditions warrant further investigation.

### Ethics approval and consent to participate

The present study was approved by the Ethical Committee on Human Experiments of Hokkaido University Graduate School of Medicine (MED08-003), and written informed consent was obtained from all subjects.

### Consent for publication

Not applicable.

## Additional files

Additional file 1: Table S1. Comparison of plasma amino acid concentrations between men and women. (DOC 45 kb) Additional file 2: Table S2. Correlations between plasma amino acid concentrations and age. (DOC 42 kb) Additional file 3: Table S3. Plasma amino acid concentrations in visceral obesity and nonvisceral obesity groups in 66 men. (DOC 46 kb) Additional file 4: Table S4. Plasma amino acid concentrations in visceral obesity and nonvisceral obesity groups in two age groups. (DOC 56 kb) Additional file 5: Table S5. Correlations between plasma amino acid concentration and indices of obesity and glucose metabolism. (XLS 19 kb) Additional file 6:
**Age- and gender-adjusted correlations between plasma amino acid concentration and indices of obesity and glucose metabolism.** (XLS 20 kb)

## Abbreviations

AA: Amino acid
VFA: Visceral fat area
SFA: Subcutaneous fat area
BCAA: Branched-chain amino acid
DM: Diabetes mellitus
IGT: Impaired glucose tolerance
BMI: Body mass index
OGTT: Oral glucose tolerance test
MRI: Magnetic resonance imaging
BW: Body weight
WC: Waist circumference
PG: Plasma glucose
NGT: Normal glucose tolerance
IFG: Impaired fasting glucose
FPG: Fasting plasma glucose
HOMA-IR: Homeostasis model assessment for insulin resistance
OGIS: Oral glucose insulin sensitivity
HOMA-β: Homeostasis model assessment of β-cell function
II: Insulinogenic index
HPLC: High-performance liquid chromatography
SPAIR: Spectral attenuated inversion recovery
GLP-1: Glucagon-like peptide-1
