# Supplementary material for: Associations among the plasma amino acid profile, obesity, and glucose metabolism in Japanese adults with normal glucose tolerance
Source: Nutr Metab (Lond). 2016 Jan 19;13:5. doi: 10.1186/s12986-015-0059-5 (PMC4717594; doi:10.1186/s12986-015-0059-5)
Supplement: Additional file 1: Table S1. — Comparison of plasma amino acid concentrations between men and women. (DOC 45 kb) [file 12986_2015_59_MOESM1_ESM.doc]

Additional file 1: Table S1. Comparison of plasma amino acid concentrations between men and women

|  | Male (*n* = 66) | Female (*n* = 17) | *p* value |
| --- | --- | --- | --- |
| **Essential AAs** |  |  |  |
| Histidine | 86.55 ± 10.12 | 81.05 ± 6.45 | **0.037** |
| Isoleucine | 66.84 ± 11.40 | 53.32 ± 8.16 | **<0.001** |
| Leucine | 131.27 ± 17.62 | 100.99 ± 12.68 | **<0.001** |
| Lysine | 192.89 ± 24.43 | 166.40 ± 23.61 | **<0.001** |
| Methionine | 26.78 ± 3.72 | 25.48 ± 3.57 | 0.200 |
| Phenylalanine | 58.55 ± 6.91 | 54.24 ± 5.15 | **0.019** |
| Threonine | 126.40 ± 23.03 | 140.21 ± 36.43 | 0.056 |
| Tryptophan | 57.12 ± 10.65 | 51.82 ± 6.04 | 0.052 |
| Valine | 240.02 ± 25.65 | 196.82 ±25.37 | **<0.001** |
| **Nonessential AAs** |  |  |  |
| Alanine | 342.41 ± 74.22 | 343.40 ± 101.01 | 0.964 |
| Arginine | 66.25 ± 15.74 | 65.53 ± 18.96 | 0.872 |
| Asparagine | 46.35 ± 6.23 | 47.02 ± 6.21 | 0.694 |
| α-ABA | 20.31 ± 5.48 | 19.34 ± 5.67 | 0.516 |
| Citrulline | 28.88 ± 6.28 | 26.26 ± 5.57 | 0.122 |
| Cystine | 37.52 ± 6.28 | 33.39 ± 6.02 | **0.017** |
| Glutamate | 62.85 ± 17.48 | 38.83 ± 12.57 | **<0.001** |
| Glutamine | 525.82 ± 57.10 | 518.27 ± 49.74 | 0.619 |
| Glycine | 218.00 ± 35.16 | 230.50 ± 32.83 | 0.189 |
| Ornithine | 82.50 ± 17.14 | 71.94 ±12.60 | **0.020** |
| Proline | 153.04 ± 40.57 | 126.86 ± 26.19 | **0.014** |
| Serine | 113.01 ± 19.35 | 129.16 ± 12.92 | **0.002** |
| Taurine | 73.84 ±19.48 | 79.46 ± 15.13 | 0.272 |
| Tyrosine | 62.43 ± 9.84 | 56.70 ±7.88 | **0.029** |

Data are mean ± SD. AA, amino acid; α-ABA, α-aminobutyric acid.
