# Supplementary material for: Associations among the plasma amino acid profile, obesity, and glucose metabolism in Japanese adults with normal glucose tolerance
Source: Nutr Metab (Lond). 2016 Jan 19;13:5. doi: 10.1186/s12986-015-0059-5 (PMC4717594; doi:10.1186/s12986-015-0059-5)
Supplement: Additional file 2: Table S2. — Correlations between plasma amino acid concentrations and age. (DOC 42 kb) [file 12986_2015_59_MOESM2_ESM.doc]

**Additional file 2: Table S2. Correlations between plasma amino acid concentrations and age**

|  | **Pearson’s correlation coefficient** | ***p* value** |
| --- | --- | --- |
| **Essential AAs** |  |  |
| Histidine | −0.017 | 0.877 |
| Isoleucine | 0.130 | 0.241 |
| Leucine | 0.089 | 0.421 |
| Lysine | 0.037 | 0.740 |
| Methionine | 0.105 | 0.344 |
| Phenylalanine | 0.073 | 0.513 |
| Threonine | 0.039 | 0.727 |
| Tryptophan | −0.117 | 0.916 |
| Valine | 0.190 | 0.086 |
| **Nonessential AAs** |  |  |
| Alanine | 0.086 | 0.439 |
| Arginine | 0.139 | 0.210 |
| Asparagine | −0.109 | 0.327 |
| α-ABA | −0.028 | 0.805 |
| Citrulline | 0.112 | 0.315 |
| Cystine | 0.141 | 0.205 |
| Glutamate | 0.220 | **0.045** |
| Glutamine | −0.254 | **0.020** |
| Glycine | −0.203 | 0.065 |
| Ornithine | 0.045 | 0.686 |
| Proline | 0.171 | 0.121 |
| Serine | −0.273 | **0.013** |
| Taurine | −0.259 | **0.018** |
| Tyrosine | 0.184 | 0.096 |

AA, amino acids; α-ABA, α-aminobutyric acid.
