# Supplementary material for: Associations among the plasma amino acid profile, obesity, and glucose metabolism in Japanese adults with normal glucose tolerance
Source: Nutr Metab (Lond). 2016 Jan 19;13:5. doi: 10.1186/s12986-015-0059-5 (PMC4717594; doi:10.1186/s12986-015-0059-5)
Supplement: Additional file 3: Table S3. — Plasma amino acid concentrations in visceral obesity and nonvisceral obesity groups in 66 men. (DOC 46 kb) [file 12986_2015_59_MOESM3_ESM.doc]

**Supplemental Table 3. Plasma amino acid concentrations in visceral obesity and nonvisceral obesity groups in 66 men**

| (nmol/ml) | **Overall (*n* = 66)** | **Visceral obesity (*n* = 18)** | **Nonvisceral obesity (*n* = 48)** | ***p* value** |
| --- | --- | --- | --- | --- |
| **Essential AAs** |  |  |  |  |
| Histidine | 86.55± 10.12 | 84.83 ± 7.94 | 87.19 ± 10.83 | 0.404 |
| Isoleucine | 66.84 ± 11.40 | 70.99 ± 10.59 | 65.29 ± 11.41 | 0.070 |
| Leucine | 131.27 ± 17.62 | 136.51 ± 15.93 | 129.31 ± 17.98 | 0.141 |
| Lysine | 192.89 ± 24.43 | 186.83 ± 21.20 | 195.16 ± 25.37 | 0.220 |
| Methionine | 26.78 ± 3.72 | 25.81 ± 3.97 | 27.14 ± 3.61 | 0.200 |
| Phenylalanine | 58.55 ± 6.91 | 59.13 ± 8.34 | 58.34 ± 6.38 | 0.680 |
| Threonine | 126.4 ± 23.03 | 122.77 ± 23.68 | 127.76 ± 22.88 | 0.438 |
| Tryptophan | 57.12 ± 10.65 | 61.94 ± 15.43 | 55.32 ± 7.64 | **0.023** |
| Valine | 240.02 ± 25.65 | 244.87 ± 25.78 | 238.20 ± 25.64 | 0.351 |
| **Nonessential AAs** |  |  |  |  |
| Alanine | 342.41 ± 74.22 | 349.52 ± 83.12 | 339.74 ± 71.36 | 0.637 |
| Arginine | 66.25 ± 15.74 | 64.26 ± 10.86 | 67.00 ± 17.26 | 0.532 |
| Asparagine | 46.35 ± 6.23 | 42.73 ± 5.92 | 47.71 ± 5.83 | **0.003** |
| α-ABA | 20.31 ± 5.48 | 20.17 ± 6.39 | 20.37 ± 5.18 | 0.895 |
| Citrulline | 28.88 ± 6.28 | 25.57 ± 4.99 | 30.12 ± 6.31 | **0.008** |
| Cystine | 37.52 ± 6.28 | 37.59 ± 7.53 | 37.49 ± 5.83 | 0.955 |
| Glutamate | 62.85 ± 17.48 | 71.88 ± 24.25 | 59.46 ± 12.92 | **0.009** |
| Glutamine | 525.82 ± 57.10 | 495.84 ± 60.17 | 537.06 ± 52.21 | **0.008** |
| Glycine | 218.00 ± 35.16 | 193.69 ± 30.16 | 227.12 ± 32.70 | **<0.001** |
| Ornithine | 82.50 ± 17.14 | 81.85 ± 15.20 | 82.75 ± 17.96 | 0.852 |
| Proline | 153.04 ± 40.57 | 151.29 ± 33.02 | 153.69 ± 43.36 | 0.833 |
| Serine | 113.00 ± 19.35 | 104.54 ± 16.36 | 116.18 ± 19.58 | **0.028** |
| Taurine | 73.84 ± 19.48 | 71.52 ± 15.75 | 74.71 ± 20.79 | 0.557 |
| Tyrosine | 62.43 ± 9.84 | 62.76 ± 9.47 | 62.31 ± 10.07 | 0.869 |

Data are mean ± SD. Unpaired *t*-tests were used for comparisons between the visceral obesity and nonvisceral obesity groups. visceral obesity:

visceral fat area ≥ 100 cm^2^, nonvisceral obesity: visceral fat area < 100 cm^2^. AA. amino acid; α-ABA, α-aminobutyric acid..
