# Supplementary material for: Associations among the plasma amino acid profile, obesity, and glucose metabolism in Japanese adults with normal glucose tolerance
Source: Nutr Metab (Lond). 2016 Jan 19;13:5. doi: 10.1186/s12986-015-0059-5 (PMC4717594; doi:10.1186/s12986-015-0059-5)
Supplement: Additional file 4: Table S4. — Plasma amino acid concentrations in visceral obesity and nonvisceral obesity groups in two age groups. (DOC 56 kb) [file 12986_2015_59_MOESM4_ESM.doc]

**Additional file 4: Table S4. Plasma amino acid concentrations in visceral obesity and nonvisceral obesity groups in two age groups**

|  | **≥35 years (*n* = 33)** | | | **<35 years (*n* = 33)** | | | |
| --- | --- | --- | --- | --- | --- | --- | --- |
| **(nmol/ml)** | **Visceral obesity**  **(*n* = 12)** | **Nonvisceral obesity (*n* = 21)** | ***p* value** | **Visceral obesity (*n* = 6)** | **Nonvisceral obesity (*n* = 27)** | ***p* value** | |
| **Essential AAs** |  |  |  |  |  |  | |
| Histidine | 86.88 ± 7.07 | 86.17 ± 9.36 | 0.820 | 80.73 ± 8.62 | 87.98 ± 11.97 | 0.172 | |
| Isoleucine | 68.16 ± 9.02 | 65.71 ± 10.06 | 0.491 | 76.67 ± 12.03 | 64.96 ± 12.54 | **0.046** | |
| Leucine | 132.57 ± 15.41 | 127.96 ± 16.35 | 0.433 | 144.40 ± 15.09 | 130.36 ± 19.39 | 0.108 | |
| Lysine | 190.20 ± 17.97 | 191.58 ± 26.59 | 0.875 | 180.08 ± 27.11 | 197.94 ± 24.51 | 0.123 | |
| Methionine | 26.93 ± 3.77 | 26.85 ± 3.87 | 0.951 | 23.57 ± 3.63 | 27.36 ± 3.45 | **0.022** | |
| Phenylalanine | 59.68 ± 8.52 | 57.34 ± 6.28 | 0.373 | 58.03 ± 8.63 | 59.11 ± 6.47 | 0.731 | |
| Threonine | 131.76 ± 22.89 | 129.43 ± 21.59 | 0.773 | 104.80 ± 13.15 | 126.46 ± 24.17 | **0.043** | |
| Tryptophan | 57.39 ± 9.19 | 54.32 ± 5.13 | 0.224 | 71.05 ± 21.78 | 56.09 ± 9.16 | **0.010** | |
| Valine | 247.81 ± 25.46 | 238.01 ± 22.67 | 0.262 | 238.98 ± 27.77 | 238.36 ± 28.16 | 0.961 | |
| **Nonessential AAs** |  |  |  |  |  |  | |
| Alanine | 376.14 ± 53.25 | 354.84 ± 58.91 | 0.309 | 296.27 ± 110.23 | 328.00 ±78.80 | 0.413 | |
| Arginine | 68.35 ± 9.96 | 65.83 ± 17.15 | 0.646 | 56.07 ± 7.87 | 67.91 ± 17.61 | 0.120 | |
| Asparagine | 44.18 ± 5.74 | 47.44 ± 6.69 | 0.167 | 39.82 ± 5.62 | 47.91 ± 5.19 | **0.018** | |
| α-ABA | 20.03 ± 4.64 | 20.31 ± 5.00 | 0.877 | 20.43 ± 9.55 | 20.41 ± 5.40 | 0.995 | |
| Citrulline | 26.47 ± 5.53 | 30.99 ± 6.88 | 0.062 | 23.77 ± 3.38 | 29.44 ± 5.87 | **0.031** | |
| Cystine | 38.87 ± 8.28 | 38.13 ± 5.13 | 0.755 | 35.03 ± 5.52 | 36.99 ± 6.38 | 0.493 | |
| Glutamate | 77.33 ± 23.33 | 63.43 ± 11.41 | **0.028** | 60.97 ± 24.23 | 56.37 ± 13.39 | 0.520 | |
| Glutamine | 485.37 ± 59.32 | 530.86 ± 54.06 | **0.032** | 516.78 ± 61.46 | 541.89 ± 51.22 | 0.302 | |
| Glycine | 197.03 ± 28.86 | 222.27 ± 37.92 | 0.055 | 187.02 ± 34.36 | 230.89 ± 28.16 | **0.002** | |
| Ornithine | 82.98 ± 14.70 | 84.10 ± 21.56 | 0.873 | 79.60 ± 17.36 | 81.69 ± 14.93 | 0.765 |  |
| Proline | 153.46 ± 29.63 | 164.25 ± 55.33 | 0.538 | 146.97 ± 41.73 | 145.48 ± 29.73 | 0.919 |  |
| Serine | 110.03 ±10.60 | 115.06 ± 19.09 | 0.409 | 93.55 ± 21.11 | 117.05 ± 20.26 | **0.016** |  |
| Taurine | 70.08 ± 15.03 | 71.58 ± 12.83 | 0.763 | 74.40 ± 18.21 | 77.14 ± 25.30 | 0.804 |  |
| Tyrosine | 65.88 ± 9.69 | 62.81 ± 8.67 | 0.356 | 56.52 ± 5.28 | 61.91 ± 11.18 | 0.261 |  |

Data are mean ± SD. Unpaired *t*-tests were used for the comparison of AA concentrations between the visceral obesity and nonvisceral obesity groups in each

age group. visceral obesity: visceral fat area ≥ 100 cm^2^, nonvisceral obesity: visceral fat area < 100 cm^2^. AA, amino acid; α-ABA, α-aminobutyric acid.
